# Disc degeneration influences the strain magnitude and stress distribution within the adjacent trabecular bone

**DOI:** 10.3389/fbioe.2024.1511685

**Published:** 2024-12-17

**Authors:** Kay A. Raftery, Alireza Kargarzadeh, Saman Tavana, Nicolas Newell

**Affiliations:** Department of Bioengineering, Imperial College London, London, United Kingdom

**Keywords:** spine, intervertebral disc, vertebra, disc degeneration, digital volume correlation

## Abstract

**Introduction:**

Up to one in five will suffer from osteoporotic vertebral fracture within their lifetime. Accurate fracture prediction poses challenges using bone mineral density (BMD) measures. Trabecular bone strains may be influenced by the underlying intervertebral disc (IVD). Understanding how disc degeneration alters load distribution to the vertebra may demonstrate that supplementing fracture risk tools with IVD metrics could improve predictions. The aim of this study was to assess the influence of IVD degeneration on the stress and strain magnitude and distribution in the trabecular bone of adjacent vertebrae.

**Methods:**

Ten human cadaveric lumbar bi-segment specimens (20 IVDs, 9 degenerated, 11 non-degenerated) were µCT-imaged under 1000N. Digital volume correlation was used to quantify axial, principal, maximum shear, and von Mises strain in the superior and inferior regions of the vertebra. Volumetric BMD from quantitative-CT was used to calculate Young’s modulus, which was then registered with the von Mises strain field to calculate internal von Mises stress.

**Results:**

Two bi-segments fractured during mechanical testing, resulting in N = 8 endplate regions per group. Trabecular bone adjacent to degenerated IVDs presented higher maximum principal and shear strains in the anterior region, relative to non-degenerated (peak ε_1_: 6,020 ± 1,633 µε *versus* 3,737 ± 1,548 µε, *p* < 0.01; peak γ_max_: 6,202 ± 1948 µε *versus* 3,938 ± 2086 µε, *p* < 0.01). Von Mises stress distribution was significantly skewed towards the anterior region in the degenerated group only (28.3% ± 10.4%, *p* < 0.05). Reduced disc height correlated with increased central-region axial compressive strain, decreased central-region BMD, and increased anterior region von Mises stress (all *p* < 0.05).

**Discussion:**

Disc degeneration may encourage high strains to be experienced within the anterior region of the adjacent bone, owing to changes in load distribution. This study demonstrates the potential of utilising IVD metrics in fracture risk assessment, to inform clinical decision making and preventative treatment.

## 1 Introduction

Vertebral compression fractures are the most prevalent type of osteoporotic fracture (Burge et al., 2007), estimated to affect 20.2% of the population over 50 years (O’Neill et al., 1996). Bone mineral density (BMD) is a surrogate of strength, typically acquired using Dual X-ray Absorptiometry (DXA). However, DXA has been criticised for its poor predictive capability, as up to 50% of post-menopausal women who sustain fractures do not present spinal osteoporosis measured using DXA (Jergas and Genant, 1997; Sanders et al., 2006). Though volumetric BMD acquired through quantitative-CT (qCT) is merited for its greater predictive accuracy relative to DXA, its true-positive rate is still only comparable to DXA-derived BMD (∼40%) at a clinically-relevant specificity (Wang et al., 2012).

In efforts to improve vertebral fracture assessment tools, it is important to address that failure is dependent on both the material properties and the loading patterns experienced, the latter of which can be assessed using internal strain measurements. At the apparent level, vertebral trabecular bone yields in compression at ∼8000 µS with minimal dependency on modulus within the anatomical site (Bayraktar et al., 2004; Kopperdahl and Keaveny, 1998; Morgan and Keaveny, 2001), and is linearly related to the experienced tissue-level strains (Bayraktar et al., 2004). Additionally, previous *in vitro* work has demonstrated that under physiological loads, high local strains in vertebral trabecular bone at the apparent level are indicative of the ultimate failure location (Tozzi et al., 2016).

The intervertebral discs (IVDs) are soft-tissue structures which act to distribute load across the endplate. It is noteworthy that 60%–80% of clinically presented vertebral compression fractures occur in tandem to endplate injury (Fujiwara et al., 2019; Ortiz and Bordia, 2011). Thus, investigating the interaction between the IVD and adjacent trabecular bone supporting the endplate may be key to delineating vertebral compression fracture initiation. It has previously been shown that loading through IVDs *versus* bone cement lowers the vertebral yield by ∼1500N (Maquer et al., 2015), and increases shear strains 2-fold in the inferior region (Hussein et al., 2013), yet the effect of disc degeneration on these strain patterns are currently unknown.

Disc degeneration is a consequence of proteoglycan matrix deterioration occurring initially in the nucleus pulposus (NP) (Pearce et al., 1987), hallmarked by disc height loss (Pye et al., 2006) and reduced intradiscal pressure (Panjabi et al., 1988). However, the effect of disc degeneration on fracture risk in the clinical literature is controversial, with evidence of both positive (Castaño-Betancourt et al., 2013; Estublier et al., 2017) and negative (Roux et al., 2008) associations found between disc degeneration and fracture incidence. *In vitro* investigations into the internal strain patterns of the bone adjacent to degenerated IVDs are needed to provide clarification on this discrepancy.

Disc degeneration and osteoporosis can simultaneously develop as a function of age; this has formed the basis of arguments that disc degeneration is associated with increased fracture risk (Geng et al., 2023; Grams et al., 2016; Kwok et al., 2012). This proposition may mean that cadaveric studies investigating the relationship between trabecular strain and disc degeneration–in which elderly specimens are commonplace–are biased. In these instances, measurement of internal stresses would supplement the interpretation of observed strain patterns by accounting for variations in the bone material properties.

Digital volume correlation (DVC) has enabled the measurement of local displacements and strains in human vertebral trabecular bone units, with the underlying IVDs present (Hussein et al., 2012; Hussein et al., 2018; Jackman et al., 2014.; Jackman et al., 2016; Palanca et al., 2023). However, DVC has not yet been harnessed to provide estimates of both the strains and stresses of bone loaded through degenerated IVDs. Paucity of information such as this perhaps explains why IVD morphological variables such as disc height are not currently used in fracture risk assessments, though it has been advocated in the past (Sornay-Rendu et al., 2004; Sornay-Rendu et al., 2006). Therefore, the aim of this study was to compare the strain, stress, and bone architecture distributions within the adjacent bone underlying degenerated and non-degenerated IVDs.

## 2 Materials and methods

### 2.1 Specimen preparation

Ethical approval was obtained from the Imperial College Tissue Bank Ethics Committee (approval number: 22/WA/0214). Ten human cadaveric L3-S1 spine segments from independent donors (Table 1) were dissected to remove all soft tissue. The facet joints were additionally removed to ensure all load was transmitted through the IVDs. Bi-segments consisting of one full vertebra (5x L4, 5x L5), two IVDs, and two-half vertebrae were sectioned, by sawing through the mid-transverse planes of the caudal and cranial vertebrae. The two half-vertebrae were potted in polymethylmethacrylate (PMMA). Sagittal- and coronal-plane fluoroscopy images (Fluoroscan Insight Mini C-arm, Hologic, Marlborough, MA) were taken to ensure that the mid-transverse plane of the middle vertebra was parallel to the pots. Prepared samples were regularly sprayed with 0.15M phosphate buffered saline (PBS) and stored at −20°C until imaging and testing, where they were thawed at 4°C for 24 h prior.

**TABLE 1:** Donor information, osteoporosis classification, and disc degeneration classification corresponding to the N = 10 samples. Osteoporosis classification was determined from the qCT BMD measurement. Pfirrmann grades (Pfirrmann et al., 2001) were calculated by averaging and rounding individual ratings from three observers.

Donor #	Cause of death	Level	Age	Gender	Donor body weight (kg)	Average vertebral CSA (mm^2^)	qCT BMD (mg/cm^3^)	Osteoporosis classification	Inferior IVD Pfirrmann grade	Superior IVD Pfirrmann grade
1	Unknown	L3-L5	39	M	77.1	1,603.3	74.9	Osteoporotic	2	3
2	Cardiopulmonary arrest, colon cancer	L3-L5	64	M	78.9	1,509.2	110.2	Osteopoenic	2	2
3	Neoplasm of Rectum	L3-L5	60	M	59.0	1,665.9	64.0	Osteoporotic	4	5
4	Cardiac arrest	L4-S1	29	F	102.1	967.1	159.9	Healthy	2	3
5	Sepsis	L4-S1	46	F	63.5	1,201.4	98.8	Osteopoenic	2	3
6	Unknown	L4-S1	50	F	62.6	1,154.7	120.6	Healthy	2	2
7	Cardiopulmonary arrest	L4-S1	68	F	103.4	1,259.3	62.3	Osteoporotic	3	3
8	Neoplasm of the Liver	L4-S1	60	M	65.3	1,568.8	59.5	Osteoporotic	2	3
9*	End Stage Epithelioid Sarcoma	L3-L5	31	M	81.6	1,568.8	51.1	Osteoporotic	3	2
10*	Astrocytoma	L3-L5	29	M	96.6	1,676.2	78.3	Osteoporotic	2	2

*Specimen fractured during testing

### 2.2 Measures of bone density and mechanical properties

To quantify volumetric BMD, axial qCT images (resolution: 0.98 × 0.98 × 1.2 mm, 110 kV, 100 mAs) of the specimens were acquired using a clinical CT scanner (IVIS SpectrumCT Imaging System, Caliper Life Sciences, Hopkinton, MA, USA). A calcium hydroxyapatite phantom (QRM GmbH, Möhrendorf, Germany) was placed underneath specimens during qCT to linearly calibrate Hounsfield units to BMD.

The trabecular bone from the middle vertebra was segmented in a commercial software (Mimics v25.0, Leuven, Belgium). Coarse exclusion of cortical bone was initially performed via patient-specific thresholding, which was then manually evaluated to dilate the trabecular volume. Segmentations were additionally reviewed for exclusion of osteophytes, and the basilar vein (Yoganandan et al., 2006). The 3D volume was meshed (3-Matic v17.0, Leuven, Belgium), where each element (side length = 2 mm) represented one BMD value derived from the scan-specific phantom calibration curve. Any additional cortical bone or spurs remaining after semi-automatic segmentation and 3D rendering were removed by thresholding at 245 mg/cm^3^ (Zhang et al., 2020) in a custom-written MATLAB script (vR 2022b, Natick, MA). Any negative BMD values (assumed to represent porous regions or air bubbles) were assigned to the lowest positive BMD value present in the volume, and a Young’s modulus of 0.0001 MPa. Assignment of these porous regions to a negligible nominal value is a method used in previous studies (Crawford et al., 2003; Jackman, DelMonaco, et al., 2016a). Donor vertebral BMD and subsequent diagnoses of bone health (healthy: BMD > 120 mg/cm^3^; osteopoenic: 80 mg/cm^3^ ≤ BMD ≤ 120 mg/cm^3^; osteoporotic: vBMD < 80 mg/cm^3^) (American College of Radiology, 2018) are reported in Table 1.

To infer vertebral trabecular bone material properties, qCT-derived density ( $\rho_{qCT}$ ) was first converted to apparent density ( $\rho_{app}$ ) using the linear relationship described by Schileo *et al.,* with an additional correction factor of 0.6 (Schileo et al., 2008). The power law relation described by Morgan *et al.* (Equation 1) was subsequently used to convert $\rho_{app}$ to Young’s modulus ( $E_{QCT}$ ) (Morgan et al., 2003):

$$E_{QCT}=4730{\rho_{app}}^{1.56}$$ (1)

Trabecular bone was assumed to behave as an isotropic and elastic material under assumptions made by infinitesimal strain theory, which applies to the trabecular bone experiencing physiological strains.

### 2.3 Disc degeneration grading and disc height measurement

T2-weighted sagittal MRIs (repetition time: 4,540 ms, echo time: 124 ms, resolution: 0.97 × 0.97 × 5 mm) of the dissected specimens were acquired on a 3T scanner (Magnetom Skyra, Siemens; Erlangen, Germany). To evaluate disc degeneration, the inferior and superior IVDs of each bi-segment were Pfirrmann graded (Pfirrmann et al., 2001) by three independent reviewers (KR, AZ, and NN). Ratings for each individual disc were averaged and rounded to obtain the final Pfirrmann grade (Table 1).

Mid-sagittal MRIs were used to measure disc height, which was calculated from the average of the posterior, central, and anterior height, as described by Bach *et al.* (Bach et al., 2019).

### 2.4 Biomechanical loading protocol and imaging for DVC

DVC relies on tracking the movement of inherent patterns between a reference and deformed body (Bay, 2008). For trabecular deformation to be tracked, µCT images (Xradia Versa 510, ZEISS; Oberkochen, Germany) of the middle vertebral body were acquired (voxel size: 0.039 mm, 140 kVp, 10W, exposure: 2 s, projections: 1801) at 50N and 1000N of axial compression. 50N was chosen as the reference state, which ensured full contact was made with the loading platen and the specimen. 1000N was chosen as the deformed state to represent a physiological compressive load when standing or walking (Rohlmann et al., 2014; Wilke et al., 1999).

**FIGURE 1:**
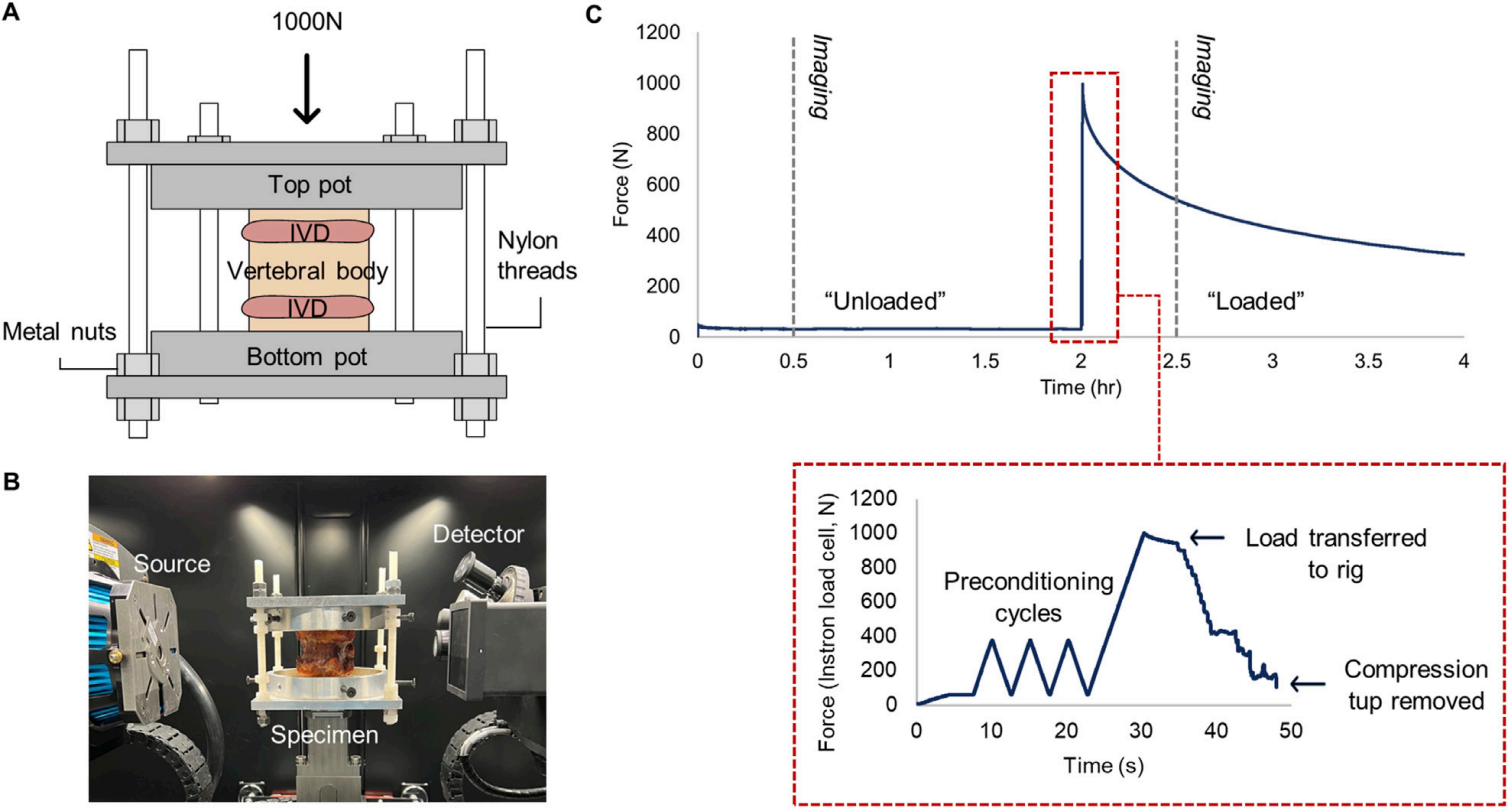
**(A)** µCT-compatible compression loading rig. The axial load was applied to the top platen, and the displacement was held by tightening the metal nuts on each nylon thread, before **(B)** placing inside the µCT chamber for image acquisition. **(C)** Force-time profile of loading and scanning protocol. Grey dashed lines indicate time of µCT acquisition. Subfigure bounded by red dashes demonstrates the procedure of load transfer to the custom rig.

Axial compression was used to minimise the interference of combined loading when interpreting strain distributions, and was achieved using a bespoke µCT-compatible compression rig (Figure 1B). Load was applied via an Instron (5,866, High Wycombe, United Kingdom) at a rate of 20% of the maximum load (N/s). After three preconditioning cycles between 50 and 300N, the displacement of the specimen was held by tightening nuts iteratively on nylon threads which connected to the top and bottom platens until the Instron load cell recorded 0N (Figure 1C). The nylon rods and nuts have been previously verified to maintain displacement with 93% accuracy when using 3D-printed platens (Rahman et al., 2023). In the present study, metal platens were used instead, thus accuracy is estimated to be higher. An inclinometer ensured the top platen was parallel with the bottom when securing the nuts. An interval of 20–30 min between displacement fixation and scanning was chosen to ensure that internal tissue deformation was negligible during imaging, as at least 87% of all stress relaxation occurs during this time (Hussein et al., 2013) (Figure 1C).

### 2.5 Microarchitectural analysis

The unloaded µCT image was used to compute trabecular thickness (Tb.Th) and trabecular separation (Tb.Sp), as these parameters together are sufficient to make inferences regarding the microarchitectural landscape. (v1.53t, Bethesda, Maryland, US) ImageJ was used for pre-processing images. A Kuwuhara filter (sampling window = 3) was applied for noise reduction. Images were then thresholded at the 89th percentile of the histogram, which corresponded to the second local trough of the bimodal distribution. Volumetric maps of Tb.Th and Tb.Sp were generated using BoneJ (Doube et al., 2010), and subsequently manually segmented to exclude cortical bone. To calculate Tb.Sp, images were down-sampled by a factor of two when using the BoneJ algorithm to reduce computational cost. It has been demonstrated that Tb.Sp is the least sensitive microarchitectural parameter to resolution changes, with a systematic error of +10% when down-sampling from 17 μm to 68 µm (Perilli et al., 2012).

### 2.6 Image pre-processing and DVC protocol

DVC was performed using DaVis (v10.2, LaVision, Germany), which employs a local approach to compute a shift vector per subset (group of voxels within the volume of interest). The vector which returns the maximum correlation coefficient, i.e., the strongest pattern overlap, is assumed to be the actual deformation of the object. Strain was derived from the optimal shift vectors using a centred finite differences scheme. A Fast-Fourier transform + direct correlation (FFT + DC) approach was chosen since FFT + DC has been shown to perform with higher accuracy relative to FFT or DC alone (Clark et al., 2020). To exclude the surrounding environment from DVC analysis, a binary mask of the volume of interest was manually segmented for each µCT reference image.

The subset size governs the strain field resolution, but compromises measurement error. A zero-strain study was performed to identify the subset size which optimised the mean absolute error (MAER) and standard deviation of the error (SDER), for each strain component of the Green-Lagrangian tensor, over the total number of subsets. An additional sample was µCT imaged twice in succession at 50N load. To find the optimal subset size, DVC was performed for 112, 96, 88, 68, 56, 38, 32, and 24 voxels (single-pass, iterations: 2), with 0% overlap between subsets (Palanca et al., 2015). A custom-written MATLAB script (Tavana et al., 2020) calculated the MAER and SDER across the endplate region. After the optimal subset size was identified, the calculation of MAER and SDER was repeated using the DVC test protocol.

Prior to performing DVC in the experimental group, rigid body movement between reference and deformed images was removed. Rigid image registration to the inferior endplate was conducted in Dragonfly (v2022.2, Object Research Systems Inc., Montreal, Canada), in which a mutual information algorithm with nearest-voxel interpolation was used to calculate a translation and rotation matrix. This matrix was applied to the deformed image in DaVis.

To perform the DVC, a predictor-corrector multi-pass scheme using the optimal subset size as the final pass was implemented (iterations: 1-2-2-3, search radius: 7 voxels, minimum valid voxel: 75%). The six Green-Lagrangian strain components were extracted and imported into MATLAB to derive axial (ε_ZZ_), maximum principal (ε_1_), minimum principal (ε_3_), and maximum shear (γ_max_) strain. Strains were omitted if the subset correlation coefficient was below 0.85, since a value above 0.8 is considered optimal for musculoskeletal applications (Dall’Ara and Tozzi, 2022).

### 2.7 Von Mises stress

To convert strains measured through DVC into stress, the von Mises strain ( $\left.\varepsilon_{VM}\right)$ was calculated as a function of all six 3D strain tensor components (Equation 2):

$$\varepsilon_{VM}=\frac{2}{3}\sqrt{\frac{3}{2}\left(\varepsilon_{xx}^{2}+\varepsilon_{yy}^{2}+\varepsilon_{zz}^{2}\right)-\varepsilon_{xx}\varepsilon_{yy}-\varepsilon_{xx}\varepsilon_{zz}-\varepsilon_{yy}\varepsilon_{zz}+3\left(\varepsilon_{xy}^{2}+\varepsilon_{xz}^{2}+\varepsilon_{yz}^{2}\right)}$$ (2)

**FIGURE 2:**
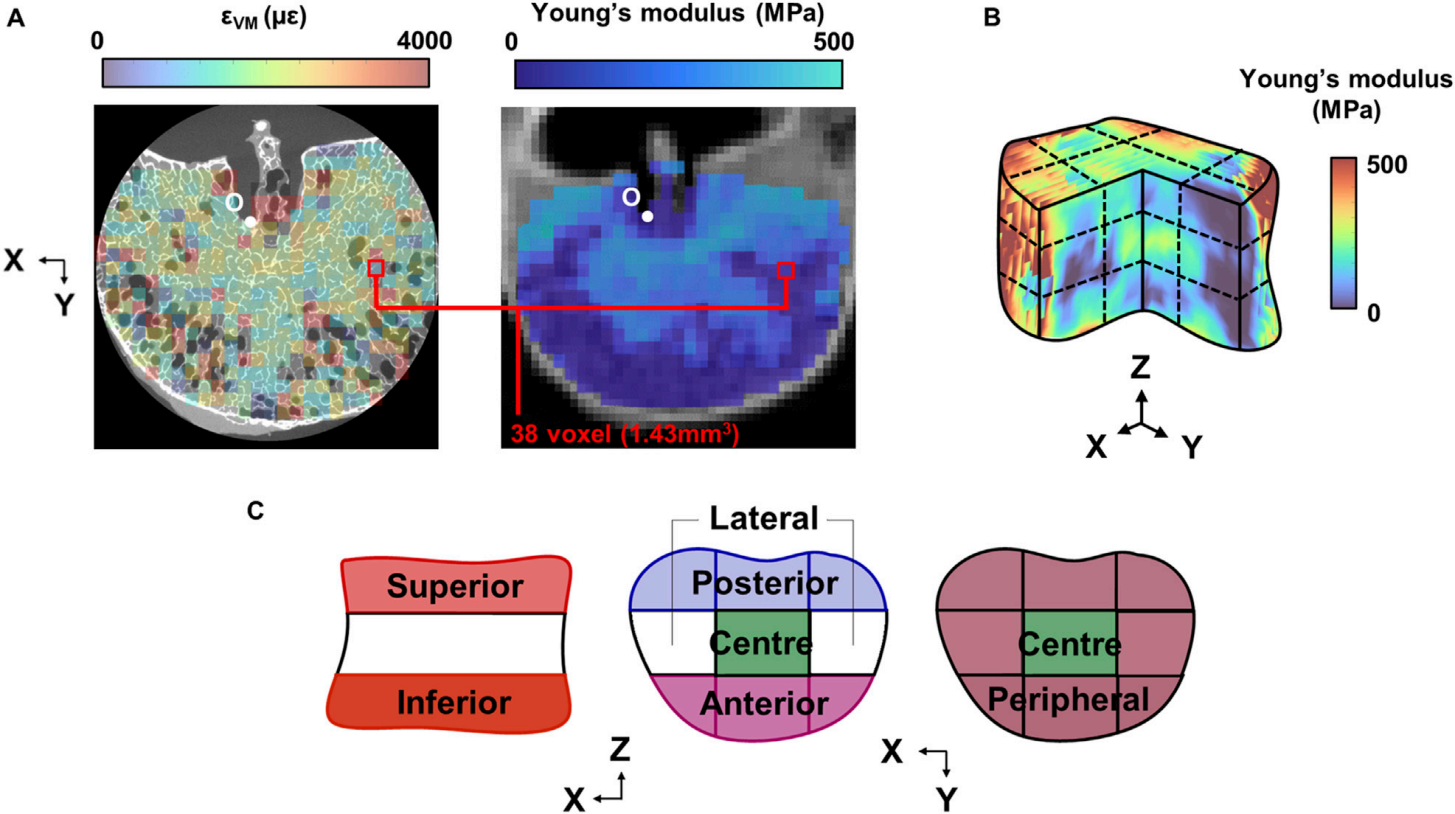
**(A)** Mid-transverse subset maps for von Mises strain and Young’s modulus for one specimen (Sample #1 shown). The maps were registered at the origin (O), defined at the 3D apex of the basilar vein. **(B)** 3D grid sectioning of the whole vertebral body, which created 27 sub-regions of which to define the **(C)** superior and inferior endplate regions, and within each endplate region, the anterior, posterior, central, lateral, and peripheral regions.

In order to register the von Mises strain field with the Young’s modulus field, the qCT images were transformed into the DaVis co-ordinate system. A custom-written MATLAB script binned the datapoints into voxels of the same dimensions as the strain subsets. The apex of the basilar vein, identifiable in both CT and µCT images, served as an anatomical landmark for the origin to be defined (Figure 2A). Due to natural lordosis and supine placement of the vertebra in the CT scanner, rigid body rotation was applied to the 3D matrix of qCT datapoints so that the local and global co-ordinate frames were aligned.

To estimate the precision associated with the von Mises stress calculation, the normalised standard error associated with the density-modulus relationship (Morgan et al., 2003), and the SDER of the strain measurement normalised to the mean von Mises strain, was combined using error propagation. Relative error was converted to absolute error using the mean von Mises stress across all samples.

### 2.8 Analysis of strain, von Mises stress, BMD, and microarchitecture

3D strain, stress, BMD, and microarchitecture fields were imported into MATLAB and were sectioned by a 9-by-9 grid (Kaiser et al., 2020) (Figure 2B). The grid lines were normalised to the two most distal X, Y, and Z co-ordinates of the whole volume. This grid was used to first section the superior and inferior thirds of the vertebra (Figure 2C), of which are hereinafter referred to as the “endplate regions”.

Within each endplate region, the volume was further divided into five sub-regions: anterior, posterior, central, peripheral, and lateral (Figure 2C). Peak strains were additionally calculated as the median of the largest 5% of values within the sub-region, to limit bias from skew or outliers. To assess the relative distribution of strain and stress within each sample, the percentage deviation between the absolute mean value of the sub-region and the whole endplate region (the mean of the nine grid volumes) was calculated.

### 2.9 Statistical analysis

Endplate regions were divided into two groups based on their Pfirrmann grade: “degenerated” (Pfirrmann grade ≥3, N = 9) and “non-degenerated” (Pfirrmann grade ≤2, N = 11). Where appropriate, groups were verified for normality using Shapiro-Wilk tests. T-tests, or Mann-Whitney tests where normality was violated, were used to compare age, disc height, and endplate region BMD between groups. 2-way repeated measures ANOVA (repeated measure: sub-region) was used to compare strain, stress, BMD, and microarchitecture, with between-groups and within-groups multiple comparison testing. The same test was performed using sub-regional strain and stress distributions as a percentage of the mean. Bonferroni post-hoc was used to account for sphericity and family-wise error. Correlations between variables were evaluated with Pearson’s r, apart from Pfirrmann grade, where Spearman’s ρ was used. A post-hoc multiple linear regression model (method: backwards elimination) verified that observed significant differences were not confounded by variables which were not matched across groups, namely: donor, age, endplate location (whether the endplate region was superior or inferior of the vertebra), vertebral cross-sectional area (CSA), and donor body weight. Alpha was set to 0.05. Statistical analyses were performed using Prism (v10.0.2, GraphPad Software, San Diego, California, United States) and SPSS (v25, IBM, Chicago, IL).

## 3 Results

Specimens #9 and #10 fractured during mechanical testing, and thus were excluded from all subsequent analyses. Resultant groups of N = 8 were matched in gender and vertebral level, and were not significantly different in age, endplate region BMD, or vertebral cross-sectional area (*p* > 0.35). Disc height in the degenerated group was significantly lower than the non-degenerated group (*p* < 0.01) (Table 2).

**TABLE 2:** Grouping of endplate regions based on adjacency to degenerated or non-degenerated IVDs. Note that fractured specimens were excluded. Significance is reported between continuous parameters (presented as mean (SD)) of the degenerated and non-degenerated groups.

	N	Superior: Inferior ratio	Male: Female ratio	L4: L5 ratio	Age	Disc height (mm)	Endplate region BMD (mg/cm^3^)	Vertebral CSA (mm^2^)
Degenerated	8	3	1	0.6	53.8 (14.2)	6.9 (1.9)	89.9 (37.9)	1,339 (261)
Non-degenerated	8	0.3	1	0.6	50.3 (12.4)	9.8 (1.2)	106.0 (30.2)	1,334 (240)
*p*					0.61	*<* 0.01 **	0.36	0.35

**p < 0.01, *p < 0.05

### 3.1 Accuracy and precision of strain and von Mises stress measurements

**FIGURE 3:**
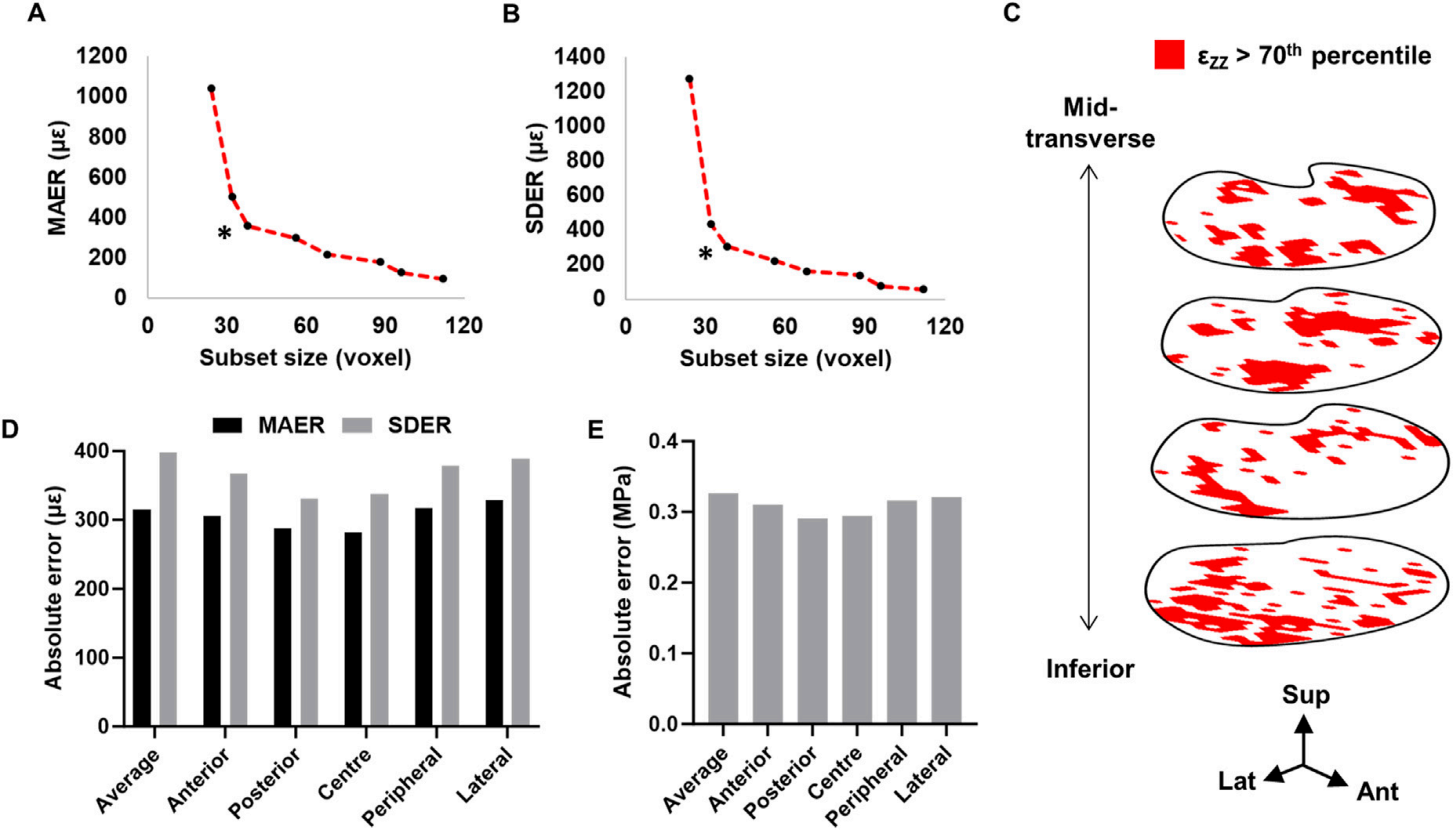
Relationship between subset size, **(A)** MAER and **(B)** SDER. * Denotes subset size used in the present study. **(C)** Distribution of >70th percentile axial strain within the inferior endplate region volume (four subset slices shown). Ant = anterior, lat = lateral, sup = superior. **(D)** MAER and SDER for each anatomical region. **(E)** Precision estimate for von Mises stress calculations.

Based on the preliminary zero-strain study, a subset size of 38 voxel (1.43 mm^3^) was chosen for its optimal trade-off between strain resolution and error (Figures 3A, B). A predictor-corrector scheme of 92–68–48–38 was subsequently used for all further analyses. In the zero-strain sample, 1.5% of the total number of subsets elicited vector shifts below the target correlation value, of which were located mostly in the posterolateral regions. Additionally, the location of high strains (>70th percentile) were randomly distributed across the volume (Figure 3C).

The mean absolute error (MAER) and standard deviation of the error (SDER) across the whole endplate region was 314.9 µε and 398.1 µε, respectively. The lateral region was highest in both MAER and SDER (328.6 µε and 388.8 µε, respectively) (Figure 3D). The precision of the von Mises stress calculation was 0.33 MPa (Figure 3E). The MAER or SDER of each grid volume did not significantly correlate with either Tb.Th or Tb.Sp (*p >* 0.32).

### 3.2 Comparison of strain and von Mises stress magnitudes

**FIGURE 4:**
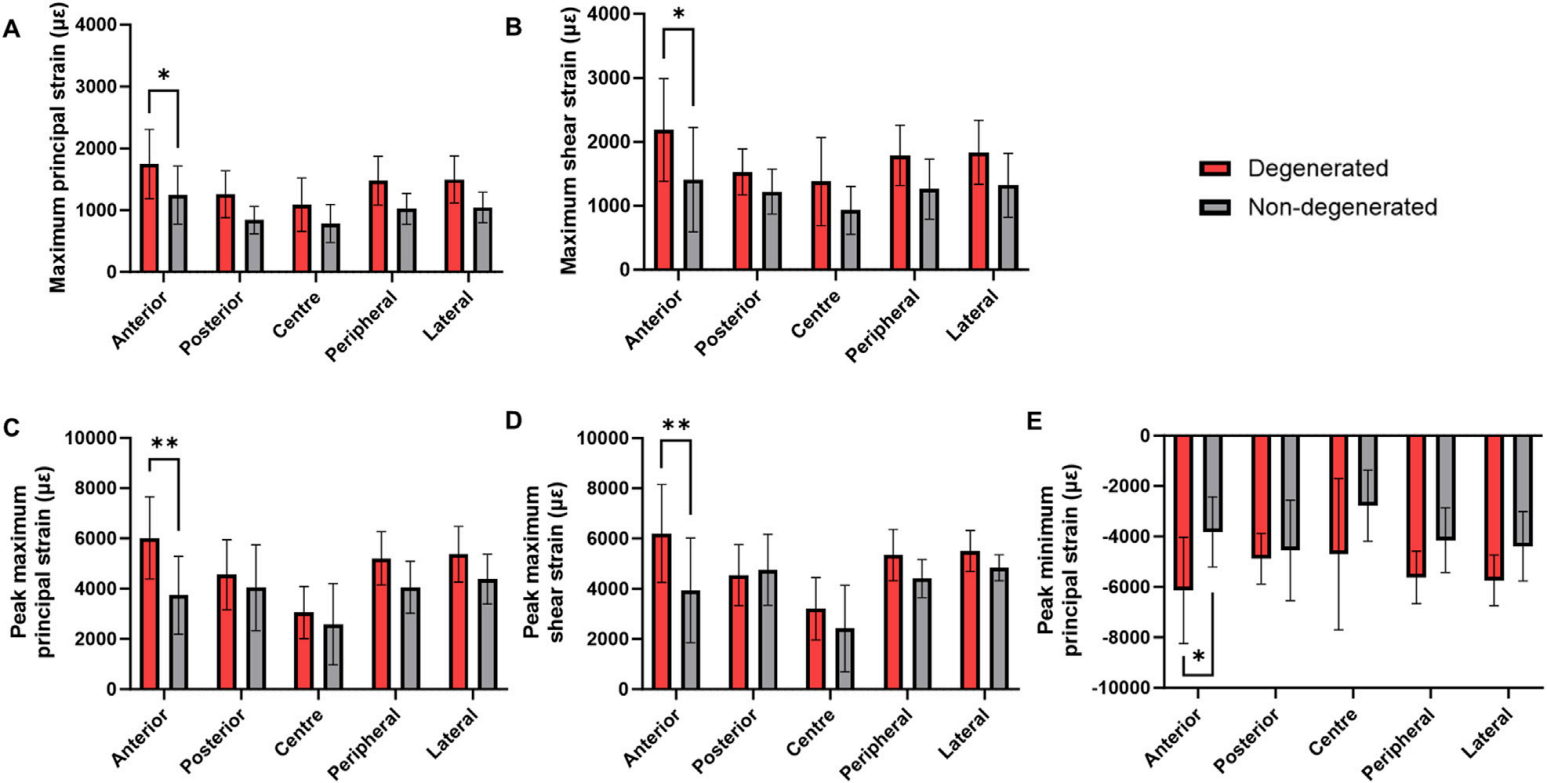
Mean (SD) **(A)** Maximum principal strain, **(B)** maximum shear strain, **(C)** peak maximum principal strain, **(D)** peak maximum shear strain, and **(E)** peak minimum principal strain within the trabecular bone adjacent to degenerated and non-degenerated IVDs. **p < 0.01, *p < 0.05.

Trabecular bone adjacent to degenerated IVDs presented significantly higher mean maximum principal strains and maximum shear strains in the anterior region relative to non-degenerated (ε_1_: 1745 ± 561 µε *versus* 1,245 ± 472 µε, *p* < 0.05; γ_max_: 2,188 ± 803 µε *versus* 1,411 ± 818 µε, *p* < 0.05) (Figures 4A, B). This pattern was similarly observed with the peak strains (ε_1_: 6,020 ± 1,633 µε *versus* 3,737 ± 1,548 µε, *p* < 0.01; γ_max_: 6,202 ± 1,948 µε *versus* 3,938 ± 2086 µε, *p* < 0.01) (Figures 4C, D). Additionally, the peak minimum principal strains were significantly higher in the anterior region of the degenerated group, relative to non-degenerated (anterior: −6,381 ± 2045 µε *versus* −4,069 ± 1775 µε, *p* < 0.05) (Figure 4E).

For each of these strain types, the corresponding linear regression model was significant (*p* < 0.05) (Supplementary Table S1), apart from the model predicting mean maximum principal strains (*p* = 0.057). Disc degeneration was the only significant predictor of changes to peak maximum principal strain (*p* < 0.05) and peak maximum shear strain (*p* < 0.01). Mean maximum shear strain was predicted by donor number and vertebral cross-sectional area (*p* < 0.05). Donor body weight was an additional predictor of anterior peak minimum principal strain (*p* < 0.05), however, presence of disc degeneration remained the strongest fitted variable (*p* < 0.01).

**FIGURE 5:**
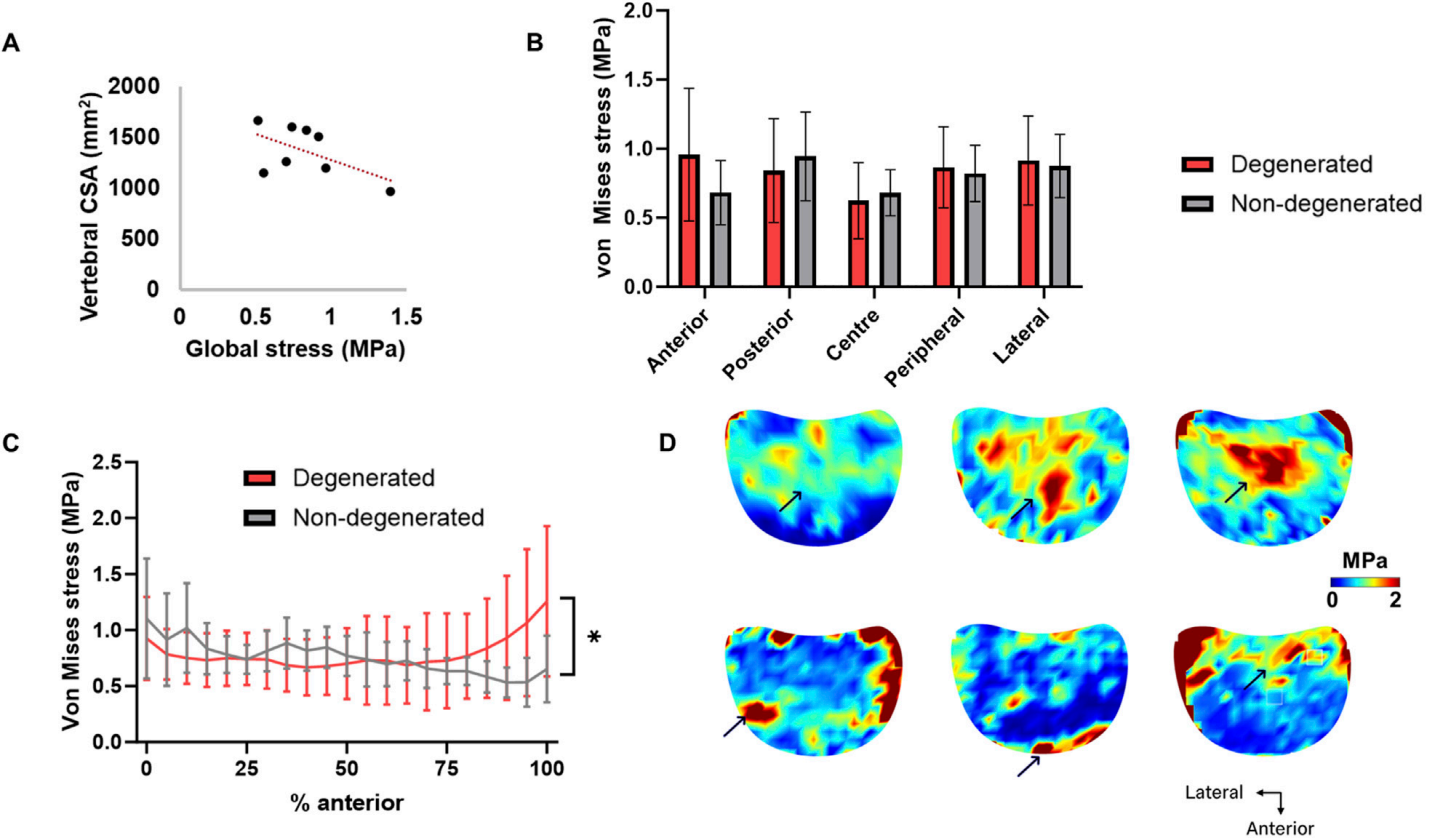
**(A)** Correlation between vertebral body global von Mises stress and cross-sectional area (CSA). **(B)** Mean (SD) von Mises stress in trabecular bone adjacent to degenerated and non-degenerated IVDs. **(C)** Magnitude of von Mises stress in the anterior-posterior plane of trabecular bone adjacent to degenerated and non-degenerated IVDs. 0% = most posterior slice, 100% = most anterior slice, where 1 slice = 1.4 mm (slice gap of one subset). **(D)** Axial von Mises stress heatmaps of three typical specimens per group. Arrows point to locations of stress concentration. *p < 0.05.

Across donors, there was an expected negative trend between vertebral body CSA and global von Mises stress, though this was not significant (r = −0.56, *p* = 0.15) (Figure 5A). Between degenerated and non-degenerated groups, the magnitude of von Mises stress was not statistically different in any region (*p* > 0.36) (Figure 5B). However, when the stress magnitudes were plotted as an average of each coronal slice, the von Mises stress was significantly higher in the most anterior region of trabecular bone underlying degenerated IVDs, relative to non-degenerated (1.256 ± 0.672 MPa *versus* 0.652 ± 0.298 MPa, *p* < 0.05) (Figure 5C). This was independent of the endplate location (*p* = 0.46). Furthermore, individual von Mises stress maps suggested that trabecular bone adjacent to degenerated IVDs typically experienced stress concentrations located in the peripheral region, whereas regions adjacent to non-degenerated IVDs experienced stress concentrations in the central region (Figure 5D).

### 3.3 Comparison of strain and von Mises stress relative distribution

**FIGURE 6:**
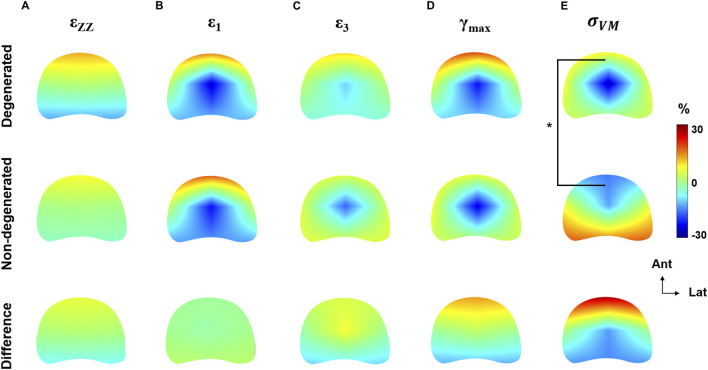
Axial heatmaps describing the distribution of **(A)** compressive axial strain, **(B)** maximum principal strain, **(C)** minimum principal strain, **(D)** maximum shear strain, and **(E)** von Mises stress normalised to the respective global mean of each endplate region. Heatmaps are plotted separately for degenerated, non-degenerated, and the difference (degenerated–non-degenerated). *p < 0.05.

The relative distribution of strain was not significantly different between degenerated and non-degenerated groups, for any strain type (Figures 6A–D). Typically, axial compressive strains were positively biased towards the anterior region (+10.9% ± 4.2%), maximum principal strains were positively biased in the anterior region (+21.9% ± 0.9%) and negatively biased in the central region (−23.8% ± 1.8%), and minimum principal strains were negatively biased in the central region (−13.7% ± 4.7%). Maximum shear strains in the degenerated group exhibited positive bias towards the anterior region (+23.0% ± 21.3%) which was less apparent in the non-degenerated group (+9.6% ± 21.3%), however this was not significant (*p* = 0.4).

However, the distribution of von Mises stress was significantly different between groups (Figure 6E), with a 28.3% ± 10.4% increase to the stress in the anterior region of degenerated endplate regions (*p* < 0.05). In the regression model, disc degeneration was the strongest fitted parameter (*p* < 0.001), but endplate location was also a significant factor (*p* < 0.01).

### 3.4 Comparison of BMD and micro-architecture

**FIGURE 7:**
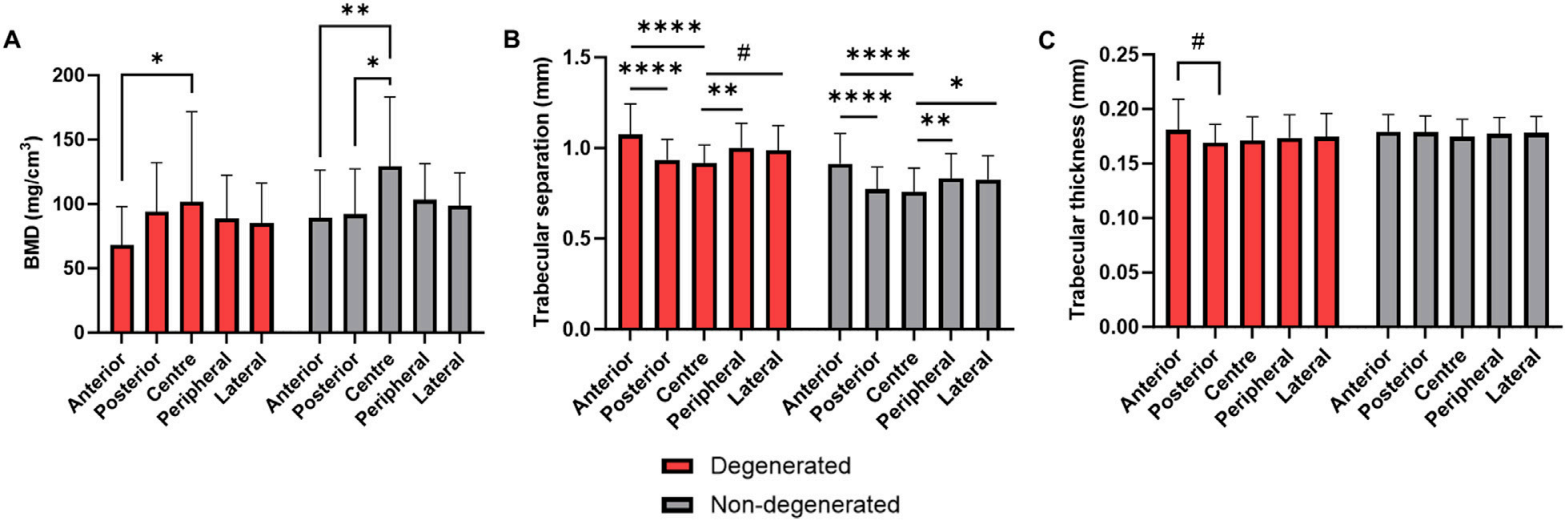
**(A)** BMD, **(B)** trabecular separation, and **(C)** trabecular thickness in endplate regions adjacent to degenerated and non-degenerated IVDs. ****p < 0.0001, ***p < 0.001, **p < 0.01, *p < 0.05, #*p* < 0.05 where effect size is smaller than the image voxel size.

There were no significant differences in BMD or microarchitecture in any region between the degenerated and non-degenerated groups (Figure 7). However, in the trabecular bone adjacent to non-degenerated endplates, central region BMD was significantly higher than both the posterior region (+37.2 ± 11.2 mg/cm^3^, *p* < 0.01) and the anterior region (+40.1 ± 11.2 mg/cm^3^, *p* < 0.01). This effect was larger than in the degenerated group, where significance was only seen between the central and anterior region (+33.8 ± 11.2 mg/cm^3^, *p* < 0.05) (Figure 7A).

Trabecular separation exhibited similar regional differences within groups, but no significant differences between groups (Figure 7B). In contrast, trabecular thickness was significantly higher in the anterior region compared to the posterior in the degenerated group only (+0.01 ± 0.003 mm, *p* < 0.05) (Figure 7C), although this effect was limited by the µCT voxel size (0.039 mm).

For all bone morphological parameters, significant differences remained when endplate regions were grouped by location.

### 3.5 Correlations with disc height and Pfirrmannn grade

**FIGURE 8:**
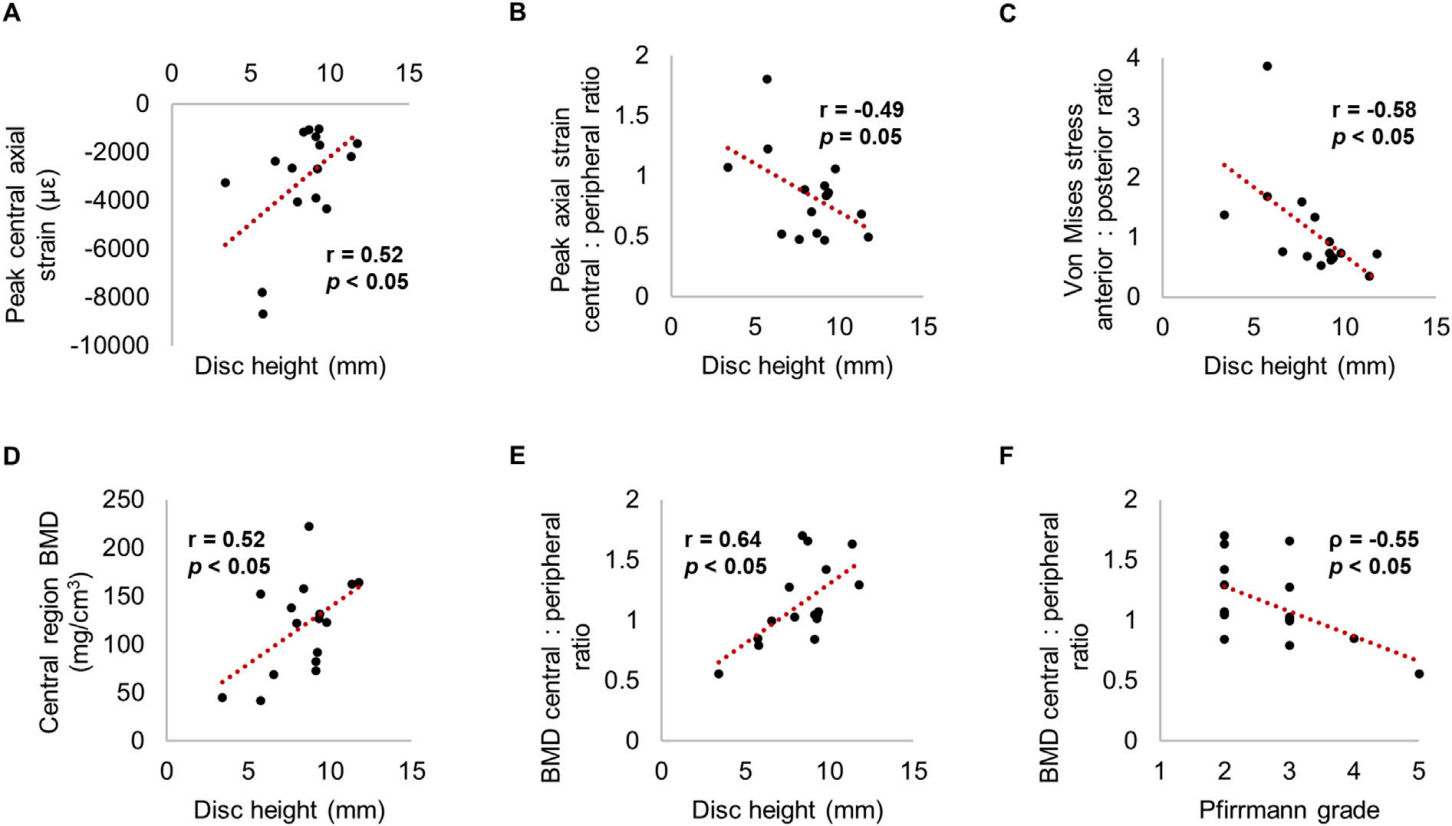
Relationships between strain, stress or BMD with **(A-E)** disc height and **(F)** Pfirrmann grade.

Decreased disc height was independently associated with increased magnitude of peak compressive axial strain and peak minimum principal strain in the central region (*p* < 0.05) (Figure 8; Table 3). Similarly, as disc height decreased, the ratio of central region compressive strain relative to the peripheral region increased (*p* = 0.05). Decreased disc height correlated with increased anterior von Mises stress relative to posterior (*p* < 0.05).

**TABLE 3:** Significant correlations between measured parameters and disc height (Pearson correlation coefficient reported) or Pfirrmann grade (Spearman rank reported). Non-significant predictors are denoted by a hyphen.

	Disc height	Pfirrmann grade
Peak central compressive axial strain	0.52	*-*
Peak central: peripheral axial strain ratio	−0.49	*-*
Peak central minimum principal strain	0.50	*-*
Posterior von Mises stress	0.58	*-*
Anterior: posterior von Mises stress ratio	−0.58	*-*
Central BMD	0.52	*-*
Central: peripheral BMD	0.64	−0.55
Central trabecular separation	−0.60	0.58
Peripheral trabecular separation	−0.56	0.63

Moreover, disc height positively correlated with central region BMD (r = 0.52, *p* < 0.05). Disc height and Pfirrmann grade correlated with the ratio between central and peripheral BMD (r = 0.64 and ρ = −0.55 respectively, *p* < 0.05); in other words, greater degeneration was associated with lower central BMD relative to the peripheral region. Increased disc height and a lower Pfirrmann grade was significantly related to decreased trabecular separation within all regions (*p* < 0.05) (Figure 8; Table 3).

## 4 Discussion

This study aimed to elucidate how disc degeneration affects the internal strains of the adjacent vertebra, whilst considering the internal stresses and morphology of the bone. This information could help to infer whether disc degeneration predisposes or protects against endplate fracture, given the temporal adaption of the trabecular bone to degeneration-induced load changes. Results demonstrate that disc degeneration was associated with increased trabecular bone strain magnitude, whilst the relative strain distribution was largely unaffected. Interestingly, the opposite was found for the internal von Mises stresses. Using clinical metrics of disc degeneration to infer which regions of the vertebra are most vulnerable to fracture may be beneficial for targeting the location of cement reinforcement in prophylactic vertebral augmentation procedures, or inform the choice and placement of instrumentation during spinal surgeries.

Maximum principal and shear strains were significantly higher in the anterior region of trabecular bone underlying degenerated IVDs, relative to non-degenerated (Figure 4). Also considering that the distribution of von Mises stress was skewed towards the anterior region (Figures 5C, 6), and that there were no changes to the anterior region BMD, Tb.Sp, or Tb.Th that were significant or discernable from the scan resolution (Figure 7), it suggests that disc degeneration encourages load to be transferred primarily through the anterior region of the adjacent trabecular bone.

This observation has failed to reach consensus in prior literature; some have reported a local increase in anterior bone quality with disc degeneration (Kaiser et al., 2020; Simpson et al., 2001; Wang et al., 2013), and greater compressive axial strains in the anterior IVD (O’Connell et al., 2011). Other works suggest as much as 28% of anterior bone is resorbed as a function of reduced disc height and increased posterior element load-bearing (Pollintine et al., 2004), and observed a 162% stress increase in the posterior region of the IVD when degenerated (Adams et al., 1996). A caveat in the present study is that the facets were removed to ensure that the load transferred to the vertebral body was controlled across samples. Furthermore, in flexion, the vertebral body is thought to tolerate ∼95% of the load, with only 6% variation due to disc degeneration (Adams et al., 2006). Thus, the loading protocol in the present study is relevant to the compression-flexion aetiology of vertebral compression fractures. Indeed, anterior shifting of high-risk tissue has been observed when modelling flexion of vertebral body specimens embedded in PMMA, but not with the boundary condition of healthy discs (Yang et al., 2012). This suggests that degenerated discs could act in a similar manner to homogenous stiff materials.

It was additionally found that central-region compressive strains increased in magnitude as disc height decreased. This was in tandem to decreased central-region BMD as disc height decreased and Pfirrmann grade increased (Figure 8). This finding compliments prior observations (Keller et al., 1989; Simpson et al., 2001), and supports the theory that disc height can be used as a surrogate of disc degeneration, whereby morphological alterations such as loss of NP hydration (Antoniou et al., 1996) and reduced intradiscal pressure (Panjabi et al., 1988) impede axial compressive force on the adjacent endplates (Jackman et al., 2014). Indeed, stress concentrations occurring in the periphery of the degenerated endplate regions, but in the centre of non-degenerated (Figure 5), highlights the difference in transmission of load from the NP to the AF (Adams et al., 1996; Jackman et al., 2016; Tavana et al., 2021). This would initially reduce the risk of bony failure, but over time manifest in trabecular bone resorption, and thus, increased strain in this region. However, correlations reported for Pfirrmann grade suggest that the lack of highly degenerated IVDs in this study could have ameliorated the magnitude of differences between groups. It is of interest to further explore the effect of Pfirrmann grade 4 and 5 IVDs in future work.

This reasoning may explain potential discordance with *in silico* studies; Polikeit *et al.* concluded a healthy disc was the “worst-case scenario” for osteoporotic vertebrae, since disc degeneration reduced the bone volume under substantial strain relative to a healthy IVD (Polikeit et al., 2004). Likewise, Homminga *et al.* reported that the volume of trabeculae at risk of fracture decreased by 50% under degenerated IVDs (Homminga et al., 2001). Similarly, greater alteration to the trabecular stress distribution, rather than the magnitude, in vertebra with underlying degenerated discs contradicts Yang *et al.*, where the opposite effect was observed upon increasing the IVD modulus (Yang et al., 2016). However, these models fail to account for the temporal remodelling of the bone occurring in a feedback loop with disc degeneration; furthermore, IVD models may neglect more nuanced markers of disc degeneration, such as NP depressurisation or heterogeneous AF strains (Tavana et al., 2021).

It is interesting to note that there were no significant regional differences between groups for BMD, trabecular thickness, and separation (Figure 7). The relationship between bone quality and strain is well established; finite element models of osteoporotic vertebra present higher principal strains under 1000N compression (Polikeit et al., 2004), and 143% greater equivalent strain in flexion/extension (Kang et al., 2022). Moreover, there is a strong body of clinical evidence pointing to a negative correlation between bone quality and disc degeneration, attributed to age-related spinal decline (Wáng, 2018). Though a similar relationship between Pfirrmann grade and global Tb.Sp was observed in this study (Table 3), relationships between Pfirrmann grade, disc height, BMD, and Tb.Sp were age-independent. This suggests that region-specific changes to bone morphology were a result of alterations to the loading distribution *in vivo,* and not solely natural ageing.

This study bears some limitations. Use of DVC for trabecular bone applications is sensitive to a low signal-to-noise ratio, which warrants caution when interpreting average regional strains in sub-failure conditions. Previous investigations using human lumbar vertebra loaded to ∼1000N (through both adjacent IVDs) have reported principal strains of 2000–4,000 µε, with a mean error as large as 700 µε (Hussein et al., 2018). Conversely, it is recommended to surpass strain error by one order of magnitude (Dall’Ara et al., 2017). For this reason, thresholding of strains at the 85th percentile or above has been advocated as a suitable evaluation technique (Oravec et al., 2021), and is suggested to provide relevant information concerning the areas most at risk of failure (Fields et al., 2010). In the present study, the standalone observations reported for peak strains (Figure 4; Table 3) support the validity of the conclusions drawn.

Secondly, both disc degeneration and endplate location were significant predictors of von Mises stress distribution, meaning it is difficult to conclude how much of this difference is attributable to disc degeneration alone. The literature has demonstrated that the superior endplate is weaker than the inferior in both clinical (Ortiz and Bordia, 2011) and biomechanical (Jackman, Hussein, et al., 2016) scenarios, owing to the weakening of the endplate and supporting trabecular structure (Zhao et al., 2009). However, the significant correlation between disc height and anterior von Mises stress relative to posterior (Figure 8) indicates a dependence on the IVD properties. Furthermore, cadaveric studies have reported no difference in fracture occurrence between superior or inferior endplates (Tavana et al., 2021), and the removal of facet joints in the present study is likely to alleviate some of the differences in behaviour between endplates.

The application of 1000N across all vertebrae may have neglected the effect of donor anthropometry on vertebral strain magnitudes, since donor body weight was an additional predictor of anterior peak minimum principal strain (Supplementary Table S1). However, a post-hoc analysis revealed that the normalisation of these strains to donor body weight did not improve the coefficient of variation (original: 42.1%; normalised: 41.2%), and the significant difference between groups was maintained (original: *p* < 0.05, normalised: *p* < 0.01), verifying the unlikelihood that anthropometrics were confounding results.

Another limitation was that it was not possible to measure mid-vertebral strains as a function of adjacent disc degeneration. If these strains propagated upwards to the mid-vertebra, this could confer a predisposition to anterior “wedge-shape” fracture, often a hallmark of vertebral compression fracture (Wáng et al., 2017). Additionally, the present study did not account for strains within the bony endplate itself. However, it is not fully elucidated how the endplate and supporting trabecular bone contribute to macroscopic failure, though risk of fracture is likely to be due to increased tensile and compressive stress, respectively (Fields et al., 2010; Yang et al., 2016). Moreover, load bearing within the superior and inferior regions of the vertebral body is largely tolerated by the trabecular bone, whereas the mid-vertebra bears a higher proportion of load through the cortical shell (Bonnheim and Keaveny, 2020; Eswaran et al., 2006). Thus, it is likely that the collapse of the underlying trabecular struts are important to initiate endplate fracture.

Lastly, registering the clinical CT and µCT images to generate the von Mises stress co-ordinate frame likely introduced subset-wise error. Only one anatomical landmark (the basilar vein) could be identified with a high enough accuracy to register the two images. However, this approach was considered superior to volumetric registration techniques, since the µCT vertebral geometries were partially obscured in order to prioritise image resolution. Moreover, the sensitivity to stress magnitude when shifting the origin by one voxel was under 6%, likely owing to the smoothing of the strain field and binning of BMD voxels to reduce local fluctuation. Given that the coefficient of variation of von Mises stress was on average 103.1%, this strengthens the conclusion that errors in this process did not appreciably contribute to the present findings.

## 5 Conclusion

This study demonstrates the potential to non-invasively measure internal stresses alongside strains in the vertebral trabecular bone, which revealed that the strain magnitudes and stress distributions in the anterior region were appreciably influenced by disc degeneration. Results contribute to the notion that supplementing vertebral fracture risk prediction with metrics of IVD morphology could ultimately optimise clinical decision-making regarding preventative and surgical treatments.

## Data Availability

The raw data supporting the conclusions of this article will be made available by the authors, without undue reservation.
