# Supplementary material for: Disc degeneration influences the strain magnitude and stress distribution within the adjacent trabecular bone
Source: Front Bioeng Biotechnol. 2024 Dec 17;12:1511685. doi: 10.3389/fbioe.2024.1511685 (PMC11685154; doi:10.3389/fbioe.2024.1511685)
Supplement: Supplementary file 1 [file Table1.docx]

| Supplementary Table 1 – Linear regression model outcomes (significant models shown only). Non-significant predictors are denoted by a hyphen | | | | | | | | | | |
| --- | --- | --- | --- | --- | --- | --- | --- | --- | --- | --- |
|  | **Individual parameters** | | | | | | | | | |
| Predictor variable | | Anterior maximum shear strain | | Anterior peak maximum principal strain | | Anterior peak minimum principal strain | | Anterior peak maximum shear strain | | Anterior von Mises stress distribution |
| Disc degeneration | | - | | < 0.05 | | < 0.01 | | < 0.01 | | < 0.001 |
| Donor number | | < 0.05 | | - | | - | | - | | - |
| Donor body weight | | - | | - | | < 0.05 | | - | | - |
| Endplate location | | - | | - | | - | | - | | < 0.01 |
| Age | | - | | - | | - | | - | | - |
| Vertebral body cross-sectional area | | < 0.05 | | - | | - | | - | | - |
|  | **Overall model** | | | | | | | | | |
| *p* | < 0.05 | | < 0.05 | | < 0.05 | | < 0.05 | | < 0.01 | |
